# Atypical Dermatitis Herpetiform and Scalp Psoriasis in Ulcerative Colitis Patient Treated With Adalimumab

**DOI:** 10.7759/cureus.35317

**Published:** 2023-02-22

**Authors:** Hamza Alshehri, Afnan Alshehri, Abrar bin Abbas

**Affiliations:** 1 Dermatology, Asir Central Hospital, Abha, SAU

**Keywords:** ulcerative colitis, adverse effect, dermatitis herpetiformis, paradoxical psoriasis, adalimumab

## Abstract

Adalimumab is a blocker of tumor necrosis factor (TNF)-alpha with established efficacy in the treatment of ulcerative colitis. However, literature indicated that adalimumab can, occasionally, induce paradoxical psoriasis reactions and, very rarely, dermatitis herpetiformis. We present a unique case of a 26-year-old female patient who developed a combination of dermatitis herpetiformis and scalp psoriasis paradoxically as a result of adalimumab treatment for ulcerative colitis. To the best of our knowledge, this is the first case of such a combination within the context of adalimumab therapy. The etiopathological underpinning of such a reaction remains elusive but is speculated to be complex and involves the interaction of several immunological and dermatological mechanisms. Adalimumab therapy is associated with a genuine risk for the development of paradoxical psoriasis and dermatitis herpetiformis. We, through this case report, added to the evidence confirming such an association. Clinicians should follow these potential adverse effects and make every effort to warn patients of their likelihood.

## Introduction

Medications that inhibit the tumor necrosis factor (TNF)-alpha, such as adalimumab, have shown effectiveness in the management of a range of inflammatory diseases, such as psoriasis, inflammatory bowel disease (IBD), and rheumatoid arthritis [1]. However, anti-TNF agents were shown to cause several adverse effects, including malignancy, increased susceptibility to infection, and malignant transformation. Counter-intuitively, anti-TNF treatment can also induce new psoriasis eruptions known as ‘paradoxical psoriasis'. Paradoxical psoriasis tends to fully disappear when anti-TNF treatment is discontinued; hence, many authors consider it a direct adverse effect of anti-TNF therapy rather than a de novo psoriasis [2]. The best estimate for the incidence of the anti-TNF therapy-induced psoriasiform eruption was 6% of all patients who started anti-TNF treatment. Moreover, the scalp and palmoplantar areas are the most affected regions with paradoxical psoriasis, and the course of the disease is usually very short [3]. 

There is no clear theoretical explanation of paradoxical psoriasis. Many authors have linked it to the predominance of innate immune-inflammatory processes in the absence of autoreactive T-cell expansion. A trickle of recent case reports has indicated that a range of immunopathological processes specific to early psoriasis was apparent in paradoxical psoriasis related to adalimumab TNF-α antagonism.

Dermatitis herpetiformis (DH) is an inflammatory dermatological disease that presents with herpetiform clusters of pruritic papulovesicular lesions. Such lesions are primarily distributed symmetrically on extensor surfaces and trunks. DH is considered a specific skin presentation of celiac disease (CD) [4].

In the present report, we discuss a case study of a 26-year-old female who, during the course of adalimumab therapy, was diagnosed with paradoxical scalp psoriasis, a recognized side effect of adalimumab. Further, she was also surprisingly diagnosed with DH as a new and unrecognized side effect of adalimumab.

## Case presentation

We present the case of a 26-year-old unmarried female patient with an established diagnosis of ulcerative colitis, started on adalimumab 160 mg subcutaneous injection. That was followed by a further 80 mg subcutaneous injection two weeks later. Then, she was prescribed a two-weekly 40 mg subcutaneous injection, which satisfactorily controlled her symptoms of ulcerative colitis.

She presented to the dermatology clinic with a two-month history of scalp lesions associated with scaling and progressive small papular lesions, coupled with itching on extremities, which started six weeks after starting the adalimumab therapy. These lesions progressed gradually. Her family and personal history were negative for similar skin conditions.

**Figure 1:**
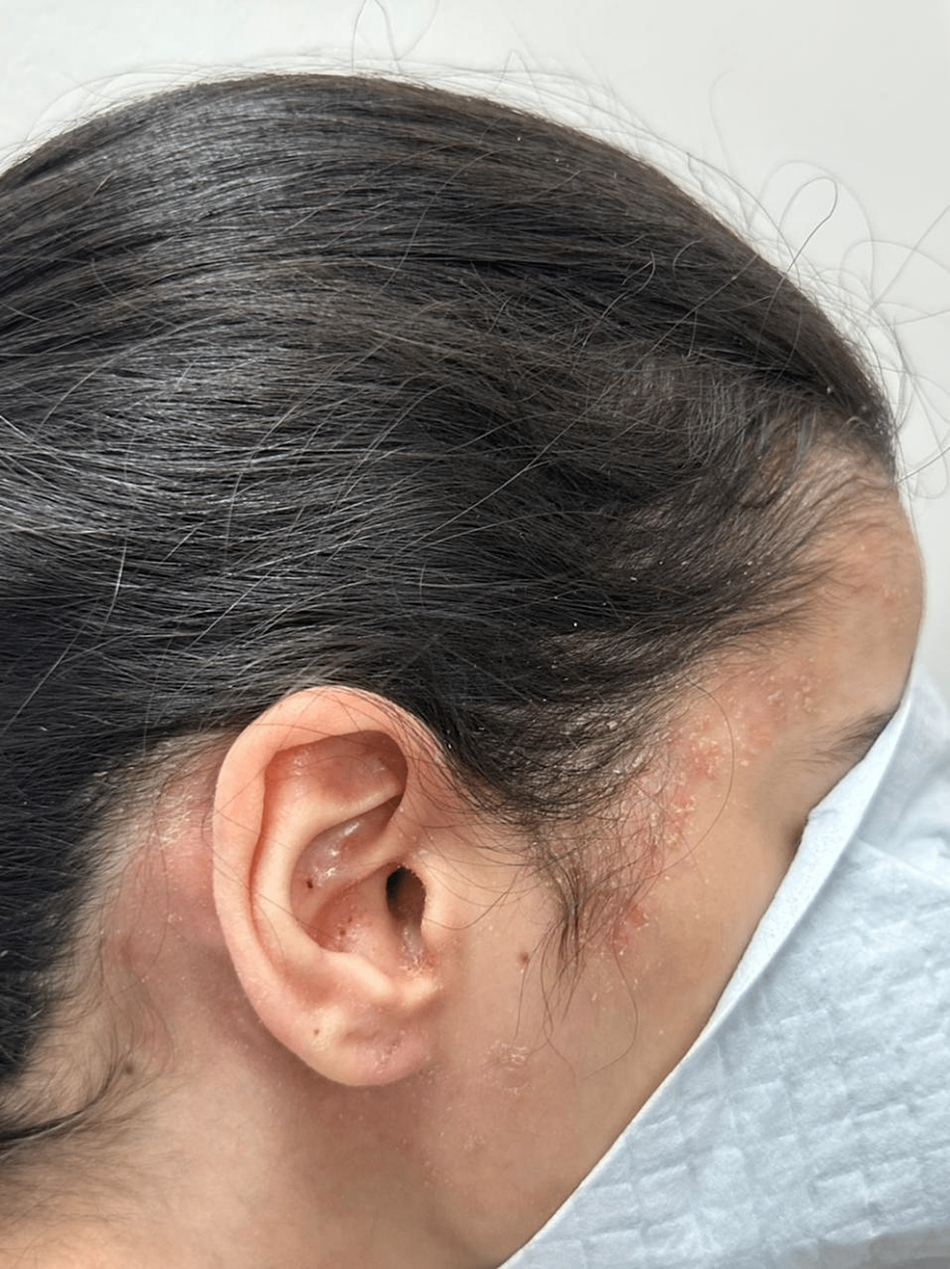
Scalp paradoxical psoriatic reaction

General physical examination results were normal. However, dermatological examination revealed well-demarcated scaly erythematous plaque overlying the scalp and hairlines extending to the periauricular area and inside the ear canal. Moreover, she had scaly plaque over her eyelids and perioral area. Figure 1 shows the pre-treatment: scalp lesions.

**Figure 2:**
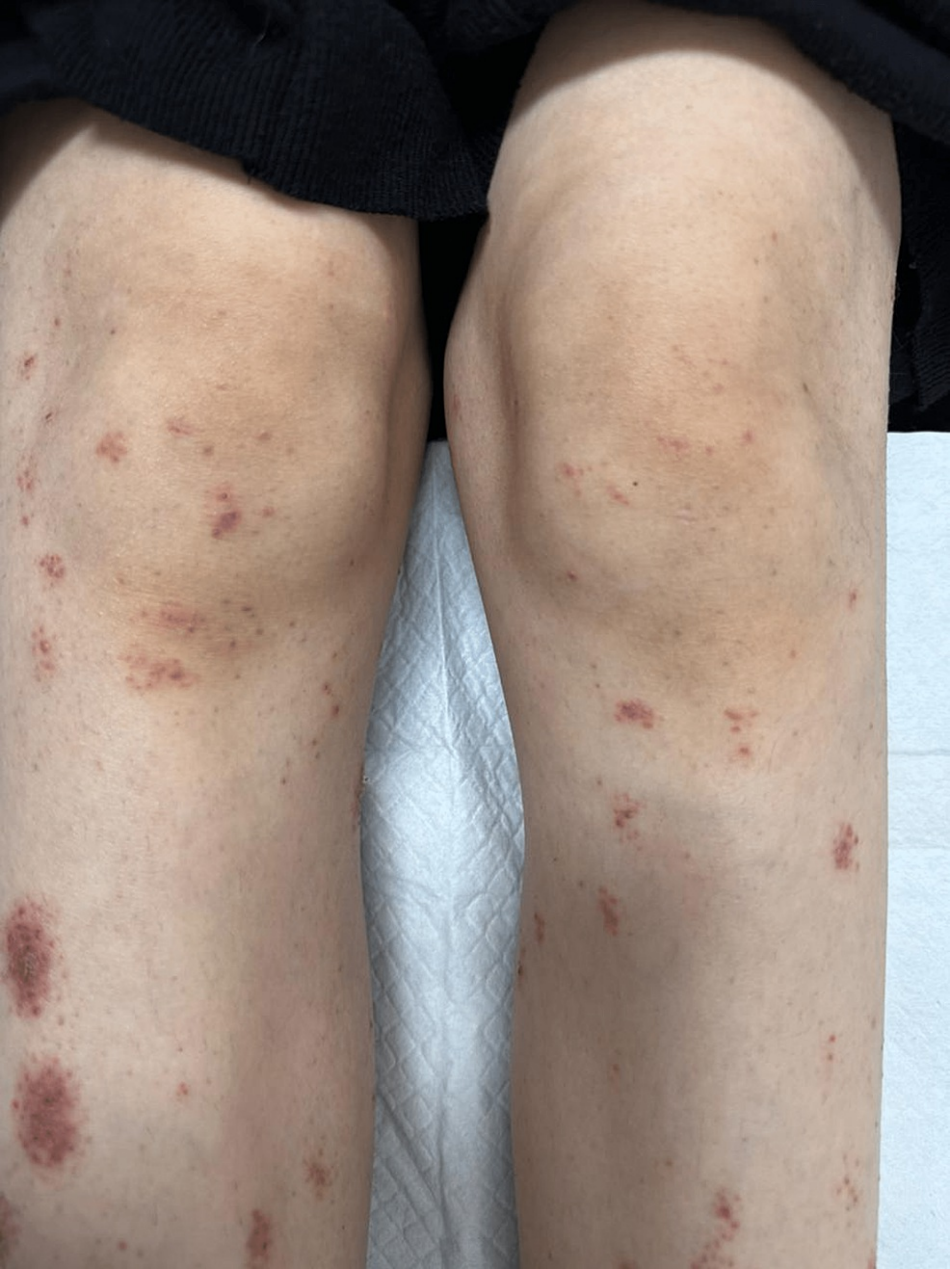
Adalimumab-induced dermatitis herpetiformis in the lower limbs

We found multiple, small, excoriated clustered papulovesicular eruptions on the erythematous base, located symmetrically over the upper and lower extremities, abdomen, genital area, and buttocks (Figure 2).

**Figure 3:**
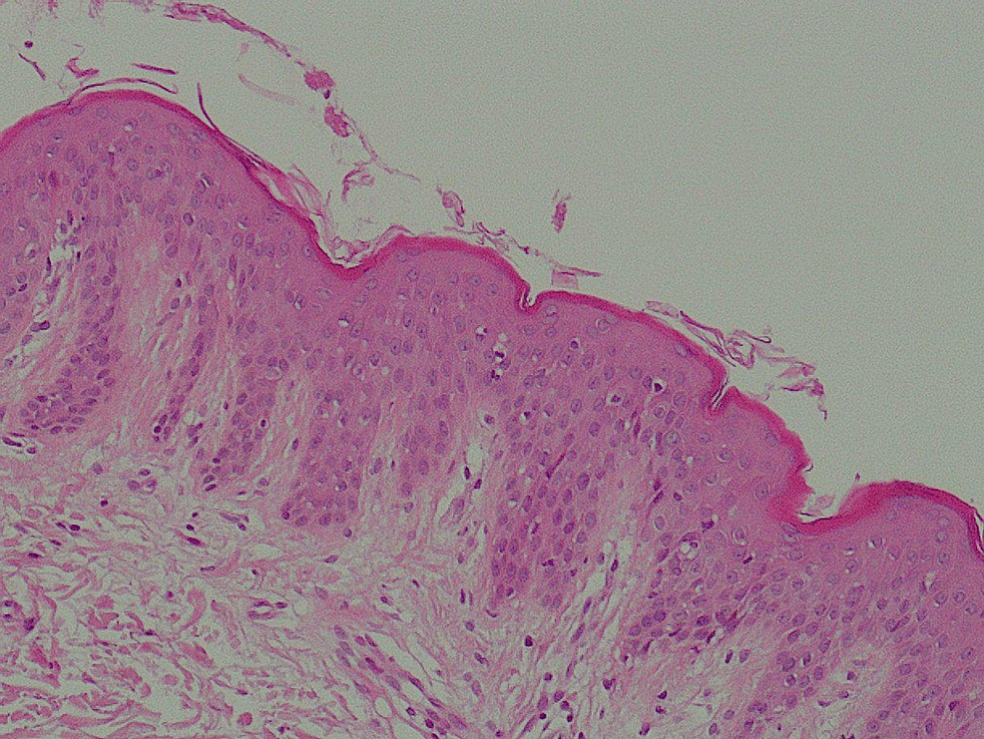
Scalp biopsy indicative of paradoxical psoriasis

Two skin biopsies were taken from the scalp and leg lesions and sent for histopathologic examination. In the scalp biopsy, there was clear evidence of hyperkeratosis, absent granular layer, regular acanthosis, tortuous dilated papillary capillaries, and transepidermal elimination of neutrophils. Such findings were consistent with scalp psoriasis (Figure 3).

**Figure 4:**
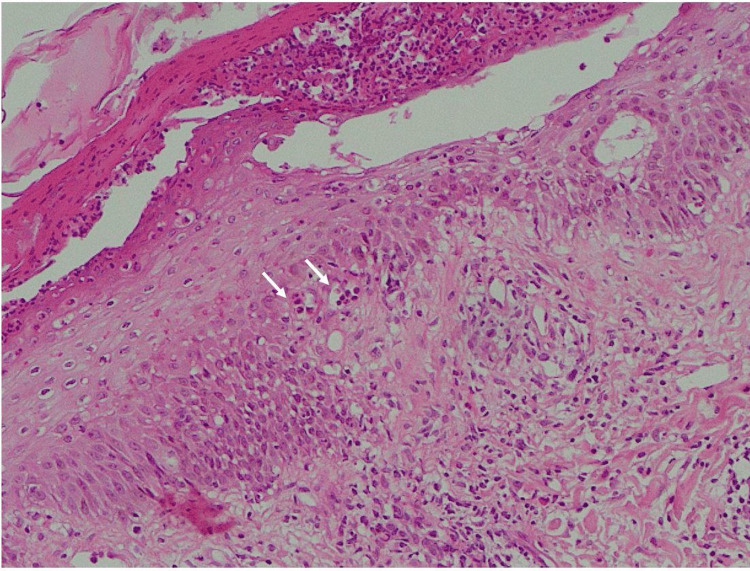
Leg biopsy indicative of dermatitis herpetiformis

The leg biopsy surprisingly showed a typical histological appearance of dermatitis herpetiform: neutrophilic collection on dermal papillae (papillary micro abscess) and neutrophilic collection at the dermo-epidermal junction (Figure 4). Direct immunofluorescence was performed and showed papillary granular IgA deposits. Unfortunately, pictures were not taken.

Laboratory tests including complete blood count (CBC), liver and renal profiles, thyroid function test, erythrocyte sedimentation rate (ESR), and C-reactive protein (CRP) were all within the normal range. We did not detect anti-human-tGA antibodies and IgA was within the normal level.

**Figure 5:**
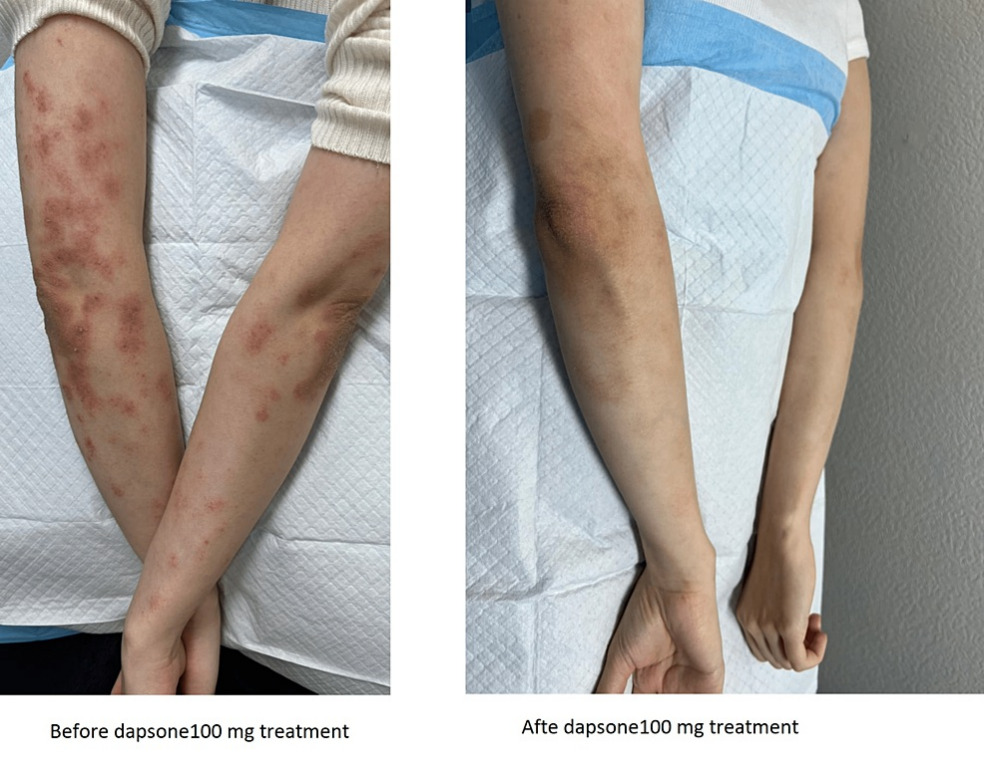
Adalimumab-induced dermatitis herpetiformis in the upper limbs pre-treatment (left) and post-treatment (right)

**Figure 6:**
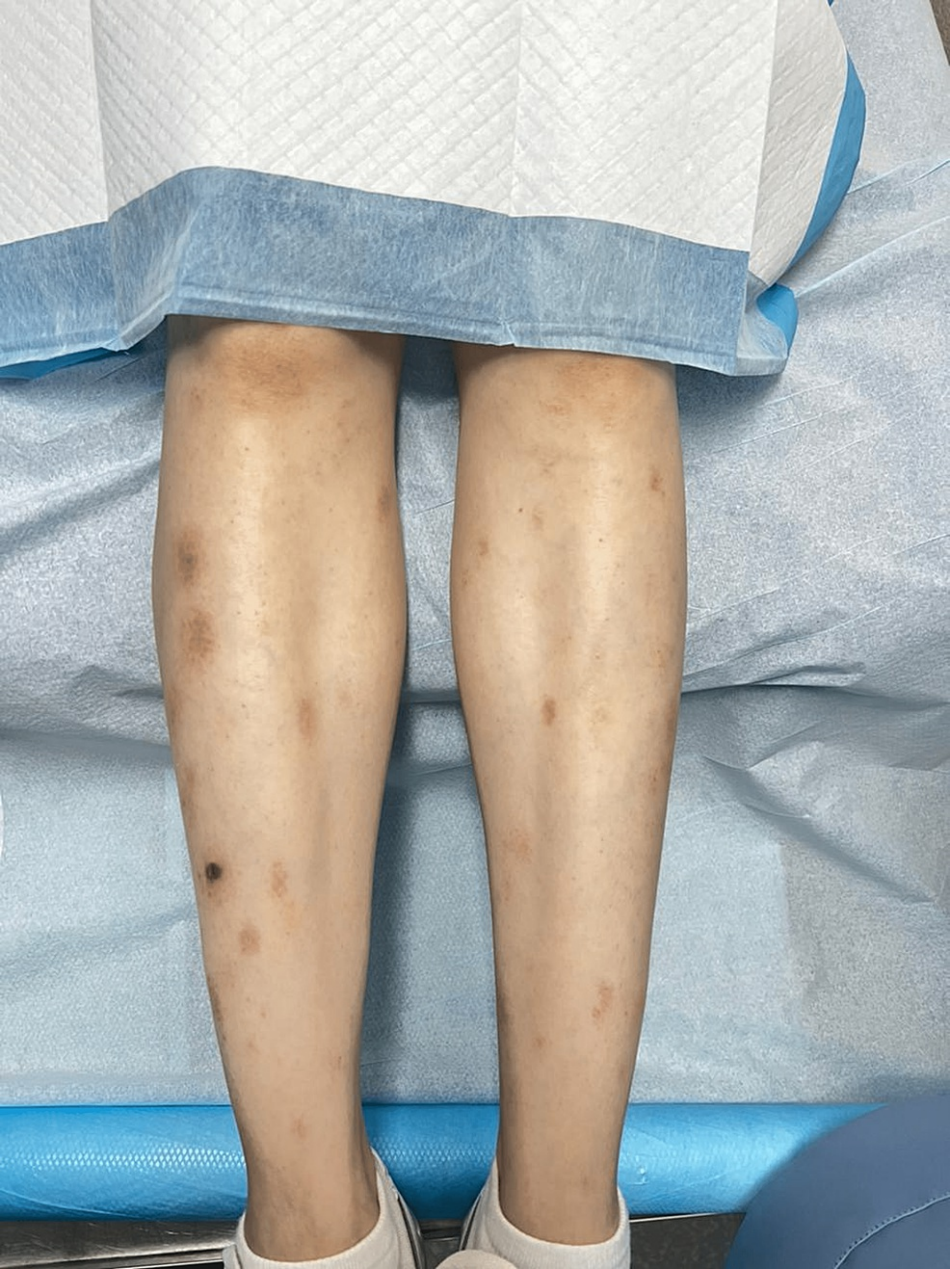
Adalimumab-induced dermatitis herpetiformis in the lower limbs (post-treatment)

Clearly, our patient paradoxically developed scalp psoriasis and DH during her treatment with adalimumab therapy. For DH, we treated the patient with a daily dose of dapsone 100 mg and after 24 hours of starting dapsone, the itching reduced significantly (Figures 5-6).

**Figure 7:**
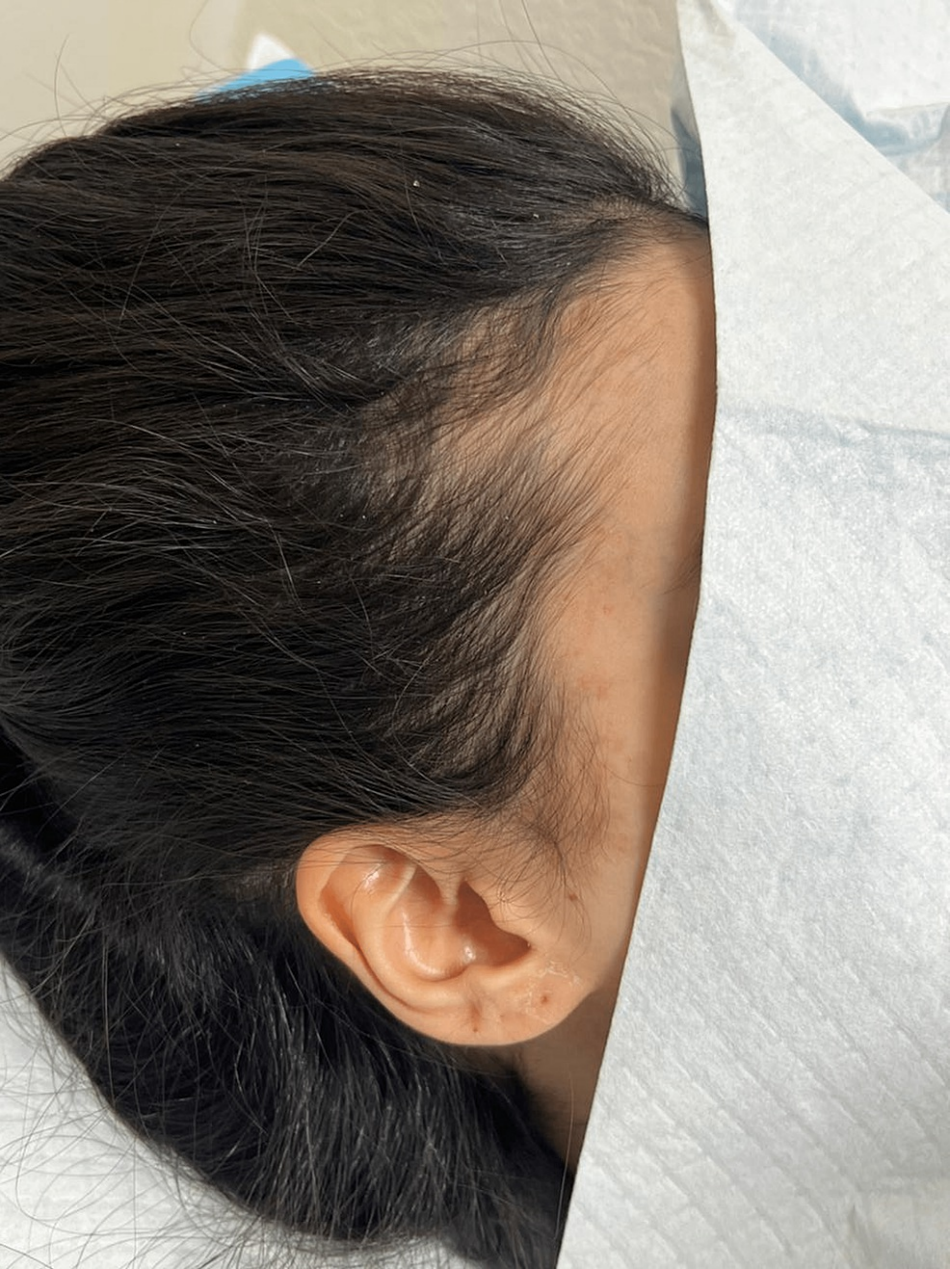
Improvement in scalp paradoxical psoriatic reaction after treatment

Regarding psoriasis of the scalp, we started on topical betamethasone valerate scalp lotion and ketoconazole shampoo 2%. After two weeks, the patient’s condition improved dramatically (Figure 7).

## Discussion

In this report, the patient, while on adalimumab therapy, paradoxically developed a combination of scalp psoriasis and DH. To the best of our knowledge, such a combination of psoriasis and DH was unheard of in the literature within the context of adalimumab treatment for ulcerative colitis. In a survey for psoriasis and DH conducted on 35 patients with rheumatic disease who were in receipt of an anti-TNF-alpha treatment course, DH was found in a single case only (a 62-year-old patient with rheumatoid arthritis on adalimumab), whereas paradoxical psoriasis was identified in 16 patients [5]. However, there are no reports of both conditions together in a single patient in the literature.

Such paradoxical psoriatic eruptions are reported more often within the context of adalimumab (and other anti-TNF-alpha agents) therapy for a range of different indications. A recent study in a couple of patients with Crohn’s disease described infliximab-induced follicular psoriasiform eruption [6]. Further, a four-month adalimumab therapy for ankylosing spondylitis was implicated in the development of severe palmoplantar pustular psoriasis. Psoriatic rashes were noted in patients on adalimumab, and it was documented as a new phenomenon since the early days of the use of the medication [7]. Similar reports were found for the development of new psoriasiform dermatitis when a course of adalimumab was commenced by a 45-year-old woman for the treatment of undifferentiated spondylarthritis [8]. However, the picture is far from a simple, direct anti-TNF-alpha-related paradoxical psoriatic reaction, as some reports indicated that some infliximab-induced psoriatic eruptions can be controlled by switching to adalimumab therapy. That led to a chain of authors trying to refute the theory regarding anti-TNF-alpha-related psoriatic reaction as a ‘class effect’ and reconsidering it as a deeper underpinning effect that can only be elucidated with further understanding of the molecular basis of psoriasis [9].

Our observation of adalimumab-induced DH is indeed unique. Only the study by Lee et al. reported such an association between adalimumab and the development of DH [5]. Given the fact that adalimumab inhibits TNF-alpha, it makes the development of DH a puzzling phenomenon, as earlier research has indicated that the activation of cutaneous endothelial skin cells was associated with increased serum levels of TNF-alpha. However, as knowledge has accumulated over the last two decades, it was apparent that etiopathogenesis of DH is quite complex and involves a wide range of intricate immunological reactions, which starts with the binding of epidermal antibodies and IgA transglutaminases, leading ultimately to matrix metalloproteinases release, inflammatory cytokine production, and neutrophil influx [10]. It is thus difficult to evaluate how adalimumab exactly affects this cascade of events, eventually leading to full-blown DH presentation.

It is, of course, difficult to make a satisfactory generalization based on our single case observation. This constitutes the primary limitation of this paper. Moreover, as we conducted no interventional analysis, our assertion of the association between adalimumab and both psoriatic reaction and DH is based on a perceived temporal relationship of events. The molecular basis for such a reaction is beyond the scope of our paper.

A further investigation of interest would be to follow patients on adalimumab for longer durations and record the development of conditions, such as DH and psoriasis, to provide a more reliable and concise estimate for the extent of both conditions. Also, molecular factors related to the development of both conditions are far from clear, and focused etiopathological research is of crucial importance to understanding why these conditions develop and how clinicians could manage them in the best possible way.

We recommend caution when commencing adalimumab therapy, as paradoxical psoriasis and DH can occur during any stage of treatment.

## Conclusions

Adalimumab therapy is associated with a genuine risk for development of paradoxical psoriasis and DH. We, through this case report, added to the evidence confirming such an association. Clinicians should follow up on these potential adverse effects and make every effort to warn patients of their likelihood to develop such conditions.
